# A Triplex Real‐Time PCR Assay for Simultaneous Detection of *Streptococcus suis*, *Glaesserella parasuis*, and *Actinobacillus pleuropneumoniae*

**DOI:** 10.1155/tbed/9983141

**Published:** 2026-05-21

**Authors:** Hongkun Zhuang, Shun Kang, Chenxu Zheng, Zeren Peng, Jinlu Zhu, Zongfu Wu

**Affiliations:** ^1^ MOE Joint International Research Laboratory of Animal Health and Food Safety, College of Veterinary Medicine, Nanjing Agricultural University, Nanjing, 210014, China, njau.edu.cn; ^2^ Key Lab of Animal Bacteriology, Ministry of Agriculture and Rural Affairs, Nanjing Agricultural University, Nanjing, 210014, China, njau.edu.cn; ^3^ WOAH Reference Lab for Swine Streptococcosis, Nanjing Agricultural University, Nanjing, 210014, China, njau.edu.cn

**Keywords:** *Actinobacillus pleuropneumoniae*, *Glaesserella parasuis*, *Streptococcus suis*, triplex real-time PCR

## Abstract

Porcine respiratory disease complex is a multifactorial disease syndrome in swine in which bacterial pathogens play important roles. Among them, *Streptococcus suis* (SS), *Glaesserella parasuis* (GPS), and *Actinobacillus pleuropneumoniae* (APP) are important bacterial agents associated with respiratory disease in pigs, underscoring the need for a rapid, accurate, and simultaneous detection method. In this study, we developed a triplex real‐time PCR assay for the simultaneous detection of SS, GPS, and APP, and evaluated its performance in tonsil samples from clinically healthy pigs. The assay showed high specificity, sensitivity, and reproducibility. A total of 228 tonsil samples were analyzed in parallel by triplex real‐time PCR assay and conventional PCR assay. The triplex real‐time PCR assay detected at least one of the three pathogens in 91.23% (208/228) of samples, which was markedly higher than the 73.68% (168/228) detected by PCR assay. Concurrent detection of multiple pathogens was observed in 98 samples (42.98%) by the triplex real‐time PCR assay, including 82 samples positive for both SS and GPS (35.96%) and 16 samples positive for SS, GPS, and APP (7.02%). From the triple‐positive samples, 12 SS isolates were recovered, of which 91.67% (11/12) were multidrug‐resistant. Animal challenge experiments further confirmed the virulence of two representative isolates, SZWUSS183 (*cps* type 1/2, ST7) and SZWUSS225 (*cps* type 3, ST117). Overall, the developed triplex real‐time PCR assay provides a sensitive and reliable tool for the simultaneous detection of these major bacterial pathogens and may facilitate surveillance of their circulation and co‐occurrence in swine herds.

## 1. Introduction

Porcine respiratory disease complex (PRDC) refers to a spectrum of respiratory diseases caused by the synergistic interplay of multiple pathogenic agents, posing a serious threat to the global swine industry and inflicting substantial economic losses [1, 2]. Among the bacterial pathogens associated with PRDC, *Streptococcus suis* (SS), *Glaesserella parasuis* (GPS), and *Actinobacillus pleuropneumoniae* (APP) are among the most prevalent and are frequently co‐detected in swine herds [3–6]. SS is a major zoonotic pathogen capable of causing meningitis, septicemia, and other systemic diseases in pigs, and it also represents a significant public health risk due to its potential to infect humans [7, 8]. GPS, formerly classified as *Haemophilus parasuis* [9], is the etiological agent of Glässer’s disease [10], which is characterized by serositis, arthritis, and meningitis [11]. APP primarily affects weaned piglets and causes porcine contagious pleuropneumonia, a disease characterized by hemorrhagic, fibrinous, and necrotizing pleuropneumonia [12].

Currently, diagnostic methods for SS, GPS, and APP rely mainly on bacterial isolation and single‐pathogen PCR assays [13]. However, co‐infections of SS, GPS, and APP are increasingly common in swine production [5, 14]. Although a multiplex PCR assay developed by Rao et al. [3] enables simultaneous detection of five pathogens, including SS, GPS, and APP, the target genes employed for the three pathogens have been shown to lack sufficient species specificity [15–17]. Thus, there is a clear need for a rapid, accurate, and simultaneous detection method for the three pathogens.

In this study, we developed a triplex real‐time PCR assay (hereafter referred to as the triplex assay) targeting the *recN* gene of SS [15], the *HPS_219690793* gene of GPS [18], and the *apx* gene of APP [19], providing a rapid and reliable tool for the simultaneous detection of these major bacterial pathogens. In parallel, bacterial isolation from triple‐positive samples was performed to further characterize the circulating SS strains, including their antimicrobial resistance profiles and virulence potential. Together, this integrated approach was designed to facilitate accurate diagnosis, epidemiological surveillance, and risk assessment of bacterial co‐detection in swine herds.

## 2. Materials and Methods

### 2.1. Bacterial Strains and Culture Conditions

All strains used in this study and their sources are listed in Table S1. GPS and APP strains were cultured in Tryptic Soy Broth (TSB) supplemented with 0.01% nicotinamide adenine dinucleotide (NAD) and 5% fetal bovine serum (FBS). After broth incubation, strains were streaked onto tryptic soy agar (TSA) plates containing 0.01% NAD and 5% FBS and then incubated at 37°C in 5% CO_2_ atmosphere. *Escherichia coli* strains were cultured in Luria‐Bertani (LB) medium. Other bacterial strains were cultured in Todd‐Hewitt Broth (THB). After broth incubation, strains were streaked onto THB agar plates and incubated at 37°C in 5% CO_2_ atmosphere.

### 2.2. Primer and Probe Design

The conserved regions of the *recN* gene of SS [15], the *HPS_219690793* gene of GPS [18], and the *apxⅣ* gene of APP [19] were obtained from the National Center for Biotechnology Information (NCBI) database (Table S1). Primers and probes were designed using Beacon Designer and Oligo 7 software. The specificity of each primer‐probe set and potential cross‐reactivity between them were analyzed using the NCBI BLAST tool and Primer Select software. All primers and probes described in this study were synthesized by Beijing Tsingke Biotech Co., Ltd., as shown in Table 1.

**Table 1:** Primers and probes used in the assay.

Assay	Primer/Probe	(5′→3′) sequence	Size (bp)
Conventional PCR assay	SS‐F	CTACAAACAGCTCTCTTCT	336
	SS‐R	ACAACAGCCAATTCATGGCGTGATT	
	GPS‐F	ACAACCTGCAAGTACTTATCGGGAT	275
	GPS‐R	TAGCCTCCTGTCTGATATTCCCACG	
	APP‐F	TGGCACTGACGGTGATGA	422
	APP‐R	GGCCATCGACTCAACCAT	

Triplex assay	triplex‐SS‐F	CAAACAGCTCTCTTCTAGCCT	175
	triplex‐SS‐R	ACATCATCGACAGTTCCACCAT	
	triplex‐SS‐P	FAM‐TGAAGATATAACCAAGCGTCTCAGCGATG‐BHQ Ⅰ	
	triplex‐GPS‐F	AACTGGCTTAGATGATTGGGACA	147
	triplex‐GPS‐R	CACGGTTCCAACATGAGCTT	
	triplex‐GPS‐P	ROX‐TGTCAAGCTATCCTGCGTTGGTCCG‐BHQ Ⅱ	
	triplex‐APP‐F	AAGCAGCCAACTCCTCAGAA	85
	triplex‐APP‐R	CAACGTCGCACAATTAATCTAAC	
	triplex‐APP‐P	VIC‐ACGCAAATCCCGAACCCGACC‐BHQ Ⅰ	

### 2.3. Preparation of Standard Plasmids

Target gene fragments were amplified by PCR using the specific primers listed in Table S2. The APP fragment was ligated into the pET‐28a (+) vector, and the SS and GPS fragments were ligated into the pMD19‐T vector. Subsequently, the resulting ligation products were transformed into chemically competent *E. coli* DH5α cells using a standard heat shock method. Positive transformants were selected on LB agar plates containing appropriate antibiotics and cultured overnight at 37°C. Recombinant plasmids were extracted using the FastPure Plasmid Mini Kit from Vazyme Biotech Co., Ltd., and their concentrations were quantified using a NanoDrop spectrophotometer. Finally, the identity and sequence accuracy of the constructed plasmids were confirmed by Sanger sequencing, which was performed by Beijing Tsingke Biotech Co., Ltd.

The plasmid concentrations were converted into copy numbers using the following formula [20]: 6.02 × 10^23^ copies/mol is Avogadro’s number, and 660 g/(mol bp) is the average molecular weight of a base pair in double‐stranded DNA (dsDNA).

amount copies/μL=DNA concentration g/μLplasmid length bp×660 g/mol⋅bp×6.02×1023copies/mol.

### 2.4. Optimization of Reaction Conditions and Establishment of Standard Curves

Equal volumes of serially diluted individual plasmid constructs were pooled and subjected to 10‐fold serial dilution, creating a standard curve with final concentrations ranging from 1 × 10^7^ to 1 × 10^3^ copies/μL. Iterative testing identified optimal reaction components in 25 μL total volume reactions, with detailed specifications provided in Table S3. Amplification was conducted on an Applied Biosystems QuantStudio 6 Flex Real‐Time PCR System (Thermo Fisher Scientific, Waltham, MA, USA). The optimized thermal cycling protocol included an initial pre‐incubation at 37°C for 30 s, followed by 40 cycles of denaturation at 95°C for 10 s and combined annealing/extension at 62°C for 30 s, with fluorescence signal acquisition during each annealing/extension step.

Method validation involved constructing a standard curve by plotting plasmid copy number concentrations (x‐axis) against the corresponding cycle threshold (*C*t) values (y‐axis). The correlation coefficient (*R* ^2^) and amplification efficiency (*E*) were calculated to evaluate the assay linearity and overall performance, confirming the reliability of the optimized triplex assay system for quantitative detection.

### 2.5. Sensitivity Test of the Triplex Assay

The sensitivity of the triplex assay was first evaluated using 10‐fold serial dilutions of mixed standard plasmids, with concentrations ranging from 1 × 10^7^ to 1 × 10^1^ copies/μL to determine the limit of detection (LOD) in accordance with the guidelines established by Bustin et al. [21]. Three gradient concentrations of plasmids (10^2^, 10^1^, and 10^0^ copies/μL) were selected for validation. Twenty replicate reactions were performed for each concentration, and the LOD was defined as the lowest concentration achieving a ≥95% positive detection rate across all replicates.

To further validate the diagnostic applicability of the triplex assay, we evaluated sensitivity across two biologically relevant sample types, including pure bacterial cultures and tonsil tissue samples. Pure cultures of SS, GPS, and APP were harvested during their logarithmic growth phase (OD_600_ = 0.6). Viable bacterial counts were quantified using the plate‐counting method, after which each culture was serially diluted with sterile phosphate‐buffered saline (PBS) to prepare template samples containing 100, 200, 400, and 500 colony‐forming units (CFUs) of the respective pathogen per 5 μL. For sensitivity in complex samples, pure bacterial cultures were spiked into SS‐negative tonsil homogenate and serially diluted with PBS to final concentrations of 100, 200, 400, and 500 CFU/5 µL. Detection was performed using these spiked tissue samples and the pure culture dilutions to compare sensitivity across matrices.

### 2.6. Specificity Test of the Triplex Assay

To evaluate the specificity of the triplex assay, pure bacterial cultures of nine bacterial pathogens (SS, GPS, APP, *Streptococcus pasteurianus*, *Staphylococcus aureus*, *Streptococcus equi* subsp. *zooepidemicus*, *Streptococcus agalactiae*, *Klebsiella pneumoniae*, and *E. coli*) from our laboratory culture collection were used as templates. Nuclease‐free water served as the non‐template negative control (NC).

### 2.7. Repeatability Test of the Triplex Assay

Intra‐assay repeatability was first evaluated using serial dilutions of standard plasmids spanning concentrations from 1 × 10^7^ to 1 × 10^4^ copies/μL, with each dilution analyzed in technical triplicate. Interassay reproducibility was assessed by performing independent replicate experiments on three separate days. Both intra‐assay and inter‐assay performance were quantitatively evaluated by calculating the coefficient of variation (CV) of *C*t values across all four concentration gradients.

### 2.8. Clinical Sample Testing

Between October 2024 and July 2025, 228 tonsil samples were collected from clinically healthy pigs at pig slaughterhouses across four regions in China, including Jiangsu (98 samples), Guangdong (92 samples), Guangxi (33 samples), and Hunan (five samples). Tonsil tissue was homogenized, and 200 μL of the homogenate was inoculated into 5 mL of THB supplemented with 0.1% NAD and 5% fetal bovine serum, followed by 8 h of enrichment prior to DNA extraction. Genomic DNA was extracted using the boiling lysis method. First, 1.5 mL of tissue homogenate enrichment broth was incubated at −80°C for 10 min and then heated at 100°C for 10 min to induce lysis and protein denaturation. After centrifugation at 12,000 rpm (revolutions per minute) for 5 min to remove cellular debris, the supernatant containing the crude DNA was collected and used directly for PCR amplification.

For a parallel assessment of detection rates, the samples were first analyzed by PCR methods for the *recN* gene of SS [15], the *HPS_219690793* gene of GPS [18], and the *apxⅣ* gene of APP [22] using previously reported primers. Reactions were conducted in 25 μL volume, containing 12.5 μL of 2 × Taq PCR Master Mix, 1 μL of the forward and reverse primers (10 μmol/L), 2 μL template DNA, and nuclease‐free water to adjust the volume. Amplification proceeded with initial denaturation at 95°C for 5 min, 35 cycles of 95°C denaturation (30 s), 55°C annealing (30 s), and 72°C extension (30 s), with a final 72°C extension for 10 min. Amplicons were resolved by 1.5% agarose gel electrophoresis (120 V, 30 min in 1 × TAE buffer), stained with GoldView, and visualized via gel imaging, and expected‐size bands indicated positive results.

All samples were subsequently analyzed using the triplex assay under the conditions specified in Section 2.4. Detection rates and co‐detection frequencies were compared between the triplex assay and conventional PCR. Since both methods were applied to the same set of samples, paired comparisons were performed using the McNemar’s test. To validate the triplex assay results and enable further pathogen characterization, two groups of samples were selected for bacterial isolation, samples that were negative by conventional PCR but positive by the triplex assay and samples that were triple positive.

### 2.9. Genomic Sequencing and Bioinformatics Analysis

SS isolated from triple‐positive samples was selected for sequencing. Genome sequencing was performed on the Illumina NovaSeq PE150 platform by Beijing Novogene Bioinformatics Technology Co., Ltd. (Beijing, China), with assembly and annotation conducted using Unicycler [23] and Prokka [24], respectively. Capsular polysaccharide (*cps*) genotyping of SS strains was determined by analyzing the *wzy* gene. Multi‐locus sequence typing (MLST) was determined by querying the PubMLST database [25]. Antibiotic resistance genes were predicted using the Comprehensive Antibiotic Resistance Database (CARD) [26]. Detailed information on all 12 SS isolates is summarized in Table 2.

**Table 2:** The information of SS isolates from triple co‐detection samples.

Strains	Accession number	*cps* type	ST	Location	Source
SZWUSS179	SAMN51603859	16	3091	Guangdong province	Clinically healthy pig
SZWUSS181	SAMN51604732	16	477	Guangdong province	Clinically healthy pig
SZWUSS183	SAMN51604882	1/2	7	Guangdong province	Clinically healthy pig
SZWUSS184	SAMN51605033	5	2254	Guangdong province	Clinically healthy pig
SZWUSS185	SAMN51605035	11	3086	Hunan province	Clinically healthy pig
SZWUSS186	SAMN51605239	10	3087	Guangdong province	Clinically healthy pig
SZWUSS202	SAMN51607993	Unknown	3088	Hunan province	Clinically healthy pig
SZWUSS203	SAMN51608110	Unknown	3089	Hunan province	Clinically healthy pig
SZWUSS204	SAMN51608134	Unknown	3090	Hunan province	Clinically healthy pig
SZWUSS225	SAMN51608251	3	117	Guangdong province	Clinically healthy pig
SZWUSS233	SAMN51608282	11	3092	Guangdong province	Clinically healthy pig
SZWUSS234	SAMN51608530	15	3093	Guangdong province	Clinically healthy pig

### 2.10. Antimicrobial Susceptibility Testing

Minimum inhibitory concentrations (MICs) were determined via broth microdilution per Clinical and Laboratory Standards Institute (CLSI) document M31‐A3 guidelines. Seventeen antibiotics representing 11 antimicrobial classes were tested, including aminoglycosides (gentamicin and spectinomycin), amphenicols (chloramphenicol and florfenicol), *β*‐lactam antibiotics (amoxicillin, cefotaxime, and penicillin), glycopeptide (vancomycin), lincosamides (clindamycin and lincomycin), macrolides (azithromycin and erythromycin), oxazolidinone (linezolid), pleuromutilin (tiamulin), quinolones (enrofloxacin), rifamycin (rifampin), and tetracyclines (tetracycline). All antibiotics were assayed at serial twofold dilutions ranging from 0.5 to 256 µg/mL. Resistance breakpoints were defined based on the CLSI document VET08‐ED4 and the European Committee on Antimicrobial Susceptibility Testing (EUCAST) guidelines.

### 2.11. Animal Infection Experiments

The virulence of strains was evaluated using zebrafish and mouse infection models. Zebrafish (15 zebrafish per group) were intraperitoneally injected with 3 × 10^6^ CFU of strains in 20 µL of PBS per zebrafish, with mortality monitored every 12 h from 12 to 96 h after the challenge. Strains exhibiting high pathogenicity (mortality rate ≥80%) were selected for validation in a mouse model. For mouse experiments, each group consisted of 10 female 5‐week‐old BALB/c mice, and each was intraperitoneally injected with 3 × 10^8^ CFU of each strain in 200 µL of PBS per mouse. The challenge doses used in both zebrafish and mouse infection experiments were determined based on our previously published studies [27–29]. Mouse survival was monitored daily over a 7‐day observation period. At the conclusion of the experiment, surviving mice were deeply anesthetized via an intraperitoneal injection of ketamine and xylazine, followed by euthanasia via cervical dislocation. Control groups in both models included the highly virulent SS serotype 2 ST7 strain SC070731 [30] and an equal volume of PBS. Survival curves across strains were compared using the Log‐rank (Mantel‐Cox) test.

### 2.12. Ethical Statements

5‐week‐old female specific pathogen‐free (SPF) BALB/c mice were purchased from Shanghai SLAC Laboratory Animal Co., Ltd. All animal experiments were carried out at the Laboratory Animal Center of Nanjing Agricultural University. The study complied with the animal welfare guidelines established by the Animal Research Committee of Jiangsu Province and was formally approved by Nanjing Agricultural University (Approval ID: NJAU.No20251027226). Anesthesia and euthanasia were carried out following the American Veterinary Medical Association (AVMA) Guidelines for the Euthanasia of Animals (2020).

## 3. Results

### 3.1. Standard Curves

**Figure 1:**
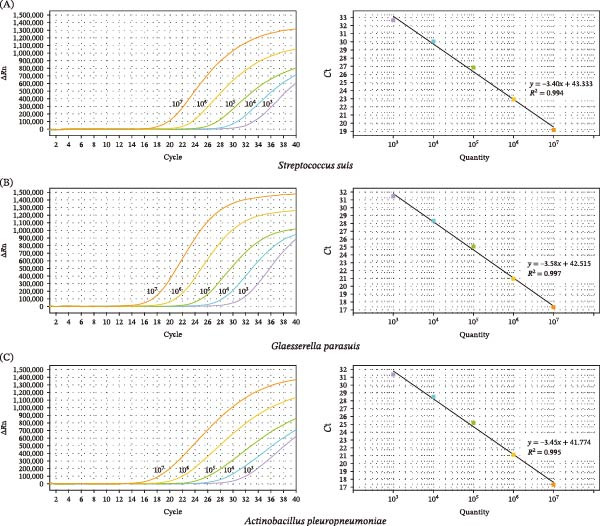
Standard curves of the triplex assay. Amplification curves (left) and standard curves (right) for (A) SS, (B) GPS, and (C) APP.

The standard curve for SS was constructed with a linear regression equation of *y* = −3.40*x* + 43.333, *R* ^2^ = 0.994, and *E* = 96.736% (Figure 1A). The standard curve for GPS was *y* = −3.58*x* + 42.515, with *R* ^2^ = 0.997 and *E* = 90.406% (Figure 1B). The standard curve for APP was *y* = −3.45*x* + 41.774, with *R* ^2^ = 0.995 and *E* = 94.942% (Figure 1C). All three standard curves exhibited high linearity (*R* ^2^ ≥0.99) and satisfactory amplification efficiencies (ranging from 90% to 110%), validating the reliability of the triplex assay for SS, GPS, and APP detection.

### 3.2. Sensitivity of the Triplex Assay

**Figure 2:**
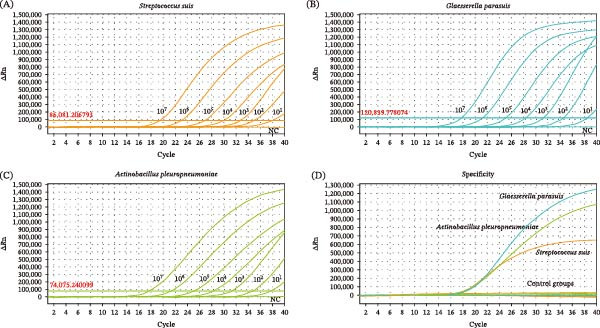
Sensitivity and specificity analysis of the triplex assay. Amplification curves of the LOD for (A) SS, (B) GPS, and (C) APP. Serial 10‐fold dilutions of mixed plasmid standards (1 × 10^1^‐1 × 10^7^ copies/μL) were tested. Amplification curves of the genomic DNA of strains in panel (D). Control groups: *S. aureus*; *S. equi* subsp. *zooepidemicus*; *S. agalactiae*; *S. pasteurianus*; *K. pneumoniae*; *E. coli*; Nuclease‐free H_2_O.

The sensitivity of the triplex assay was evaluated using positive plasmids spanning concentrations from 1 × 10^7^ to 1 × 10^1^ copies/μL. Combined analysis of sensitivity assays (Figure 2A–C) and the LOD determination experiments (Table S4) confirmed that the triplex assay achieved a consistent positive detection rate of ≥95% for the target gene at the lowest concentration of 1 × 10^2^ copies/μL, establishing a minimum LOD of 1 × 10^2^ copies/μL for SS, GPS, and APP. These findings validate the high sensitivity of the established triplex assay, confirming its reliability for detecting target genes at low copy number levels.

For pure bacterial cultures, the LOD of the triplex assay for SS, GPS, and APP was established as 200 CFU, 200 CFU, and 400 CFU, respectively. In tonsil tissue samples, the LOD for all three pathogens was consistent at 500 CFU. These results demonstrated that the method exhibited excellent detection performance in complex sample matrices.

### 3.3. Specificity of the Triplex Assay

To further assess the potential cross‐reactivity of the triplex assay, pure bacterial cultures of six nontarget pathogens were prepared, including *S. pasteurianus*, *S. aureus*, *S. equi* subsp. *zooepidemicus*, *S. agalactiae*, *K. pneumoniae*, and *E. coli*. No nonspecific amplification was detected in any of these non‐target pathogen templates; specific amplification signals were observed exclusively for SS, GPS, and APP, as illustrated in Figure 2D.

### 3.4. Repeatability of the Triplex Assay

The intra‐assay repeatability and inter‐assay reproducibility of the triplex assay were assessed by calculating the CVs of *C*t values. These values were derived from triplicate amplifications of mixed standard plasmids with concentrations spanning from 1 × 10^7^ to 1 × 10^4^ copies/μL. The intra‐assay CVs ranged from 0.48% to 1.58%, while inter‐assay CVs ranged from 0.60% to 2.03%, with detailed data presented in Table S5. These results confirm that the established triplex assay exhibits excellent repeatability and reproducibility, supporting its reliability for consistent quantitative detection.

### 3.5. Clinical Sample Detection and Comparison With Conventional PCR

**Figure 3:**
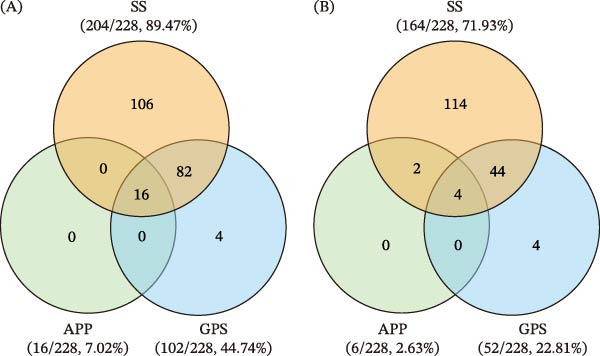
Comparison of detection results for SS, GPS, and APP using triplex assay and conventional PCR assay. (A) Detection rates obtained with triplex assay. (B) Detection rates obtained with conventional PCR assay. Values in circles indicate the corresponding number of positive samples. Co‑detection patterns are indicated within the overlapping regions of the diagrams.

A total of 228 tonsil samples from clinically healthy pigs in four Chinese provinces were tested in parallel using the triplex assay and conventional PCR. The triplex assay detected at least one pathogen in 208 samples (91.23%), whereas conventional PCR detected at least one pathogen in 168 of 228 samples (73.68%) (*p* < 0.001). The detection rates of the triplex assay were 89.47% (204/228) for SS, 44.74% (102/228) for GPS, and 7.02% (16/228) for APP (Figure 3A), compared with 71.93% (164/228), 22.81% (52/228), and 2.63% (6/228), respectively, for conventional PCR (Figure 3B). The differences between the two methods were statistically significant for SS (*p* < 0.001), GPS (*p* < 0.001), and APP (*p* < 0.005). Regarding co‐detection, the triplex assay identified 98 co‐detection cases (98/228, 42.98%), among which SS‑GPS dual detection was the most common (82/228, 35.96%), followed by SS‑GPS‑APP triple detection (16/228, 7.02%). In contrast, the PCR assay detected only 50 co‐detection cases (50/228, 21.93%). To further support the reliability of the triplex assay, bacterial isolation was attempted from tonsil samples that were negative by conventional PCR but positive by the triplex assay. Six SS strains and two GPS strains were isolated as listed in Table S6. These results support the higher sensitivity and reliability of the triplex assay developed in this study.

### 3.6. *cps* Type and MLST Typing of SS Isolates

Twelve SS isolates were recovered from the 16 triple‐positive tonsil samples. Among them, four isolates (SZWUSS185, SZWUSS202, SZWUSS203, and SZWUSS204), belonging to different *cps* and ST types, were obtained from a single tonsil sample, while each of the other eight positive samples yielded one isolate. The remaining seven triple‐positive samples did not yield any isolates upon culture. The *cps* types and MLST profiles of these isolates are detailed in Table 2. All isolates were assigned to 12 distinct sequence types (STs), encompassing seven *cps* types and 3 nontypeable strains. Notably, two isolates exhibited specific combinations of *cps* and ST types, which warrant further investigation in subsequent virulence investigations. SZWUSS183 was classified as *cps* type 1/2 and ST7, while SZWUSS225 belonged to *cps* type 3 and ST117.

### 3.7. Antimicrobial Susceptibility Profiles of SS Strains

**Figure 4:**
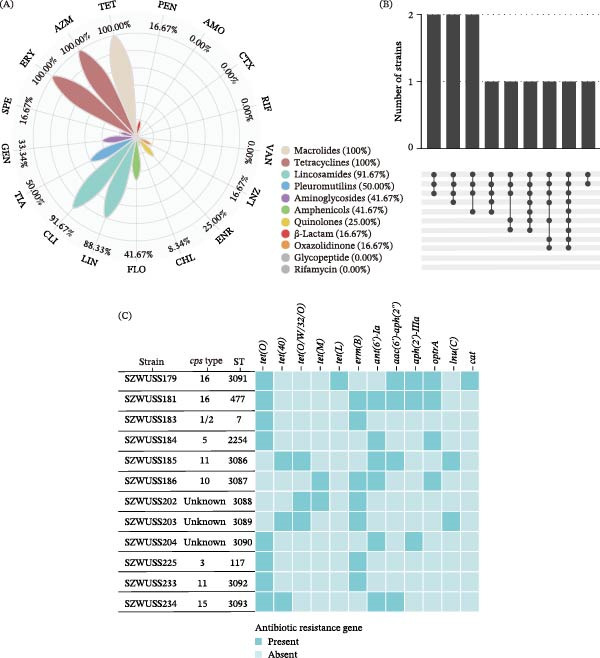
Antibiotic resistance characteristics of SS isolates. (A) The resistance rates of 12 strains to 17 antibiotics. AMO, amoxicillin; AZM, azithromycin; CHL, chloramphenicol; CLI, clindamycin; CTX, cefotaxime; ENR, enrofloxacin; ERY, erythromycin; FLO, florfenicol; GEN, gentamycin; LIN, lincomycin; LNZ, linezolid; PEN, penicillin; RIF, rifampin; SPE, spectinomycin; TET, tetracycline; TIA, tiamulin; VAN, vancomycin. (B) The resistance rates of 12 strains to 11 categories of antibiotics. (C) Antimicrobial resistance gene profiles of the 12 SS isolates.

As presented in Figure 4A, all isolates demonstrated resistance to erythromycin, azithromycin, and tetracycline. More than 80% of the isolates exhibited resistance to clindamycin (91.67%, 11/12) and lincomycin (83.33%, 10/12). Resistance rates to tiamulin, florfenicol, gentamicin, enrofloxacin, spectinomycin, penicillin, linezolid, and chloramphenicol were 50.00%, 41.67%, 33.34%, 25.00%, 16.67%, 16.67%, 16.67%, and 8.34%, respectively. All isolates were susceptible to amoxicillin, cefotaxime, rifampin, and vancomycin. Notably, 91.67% (11/12) of the isolates were resistant to ≥3 classes of antimicrobial classes (Figure 4B), qualifying them as multidrug‐resistant (MDR) strains. The MIC values for all 17 tested antimicrobials are detailed in Table S7.

The antibiotic resistance gene profiles of the 12 SS isolates are shown in Figure 4C. Twelve unique resistance genes were detected, belonging to functional categories including tetracycline, macrolide‐lincosamide‐streptogramin B (MLS_B_), aminoglycoside, lincosamide, oxazolidinone, and chloramphenicol resistance determinants. Tetracycline resistance genes were ubiquitous (100%, 12/12), matching the observed tetracycline resistance phenotype. Among these, *tet*(*O*) was the most prevalent (66.67%, 8/12), followed by *tet*(*O/W/32/O*) (25.00%, 3/12), *tet*(*40*) (25.00%, 3/12), *tet*(*M*) (16.67%, 2/12), and *tet*(*L*) (8.33%, 1/12). Aminoglycoside resistance genes were detected in 58.33% (7/12) of isolates, including *ant*(*6*)*-Ia* (50.00%, 6/12), *aac*(*6*′)*-aph*(*2*″) (33.33%, 4/12), and *aph*(*3*′)*-III* (25.00%, 3/12). The MLS_B_ resistance gene *erm*(*B*) was carried by 58.34% (7/12) of isolates, correlating with their resistance to erythromycin, azithromycin, lincomycin, and clindamycin. Additionally, the lincosamide resistance gene *lnu(C)* was detected in 16.67% (2/12) of the isolates. The *optrA* gene, which mediates resistance to florfenicol and linezolid, was present in 33.33% (4/12) of isolates, whereas the chloramphenicol resistance gene *cat* was identified in 8.33% (1/12).

### 3.8. Animal Infection Experiments

As summarized in Table 3, two isolates (SZWUSS183 and SZWUSS225) showing 80% mortality in zebrafish were classified as virulent. To further validate the virulence, a BALB/c mouse infection model was employed using these two isolates along with two low‐virulence strains (<40% zebrafish mortality). Results from the mouse challenge (Table 4) showed that SZWUSS183 caused 80% mortality, with survival curves exhibiting no significant difference from the serotype 2 ST7 virulent control strain SC070731, confirming its high virulence. SZWUSS225 resulted in a 70% mortality. In contrast, the two low‐virulence strains failed to induce any mortality in mice. Collectively, these findings confirm the pathogenic potential of these two clinical isolates.

**Table 3:** The results of the zebrafish infection experiment.

Strain	*cps* type	Deaths at different post‐infection time points								Total deaths	Mortality rate (%)	*p* value	Significance^a^
		12 (h)	24 (h)	36 (h)	48 (h)	60 (h)	72 (h)	84 (h)	96 (h)				
SC070731	2	8	7	0	0	0	0	0	0	15	100.00	—	—
PBS	—	0	0	0	0	0	0	0	0	0	0.00	—	—
SZWUSS179	16	1	0	1	1	0	2	0	0	5	33.33	<0.0001	^∗∗∗∗^
SZWUSS181	16	2	0	0	1	0	1	0	0	4	26.67	<0.0001	^∗∗∗∗^
SZWUSS183	1/2	4	5	3	0	0	0	0	0	12	80.00	0.0105	^∗^
SZWUSS184	5	0	3	2	0	0	1	0	3	9	60.00	<0.0001	^∗∗∗∗^
SZWUSS185	11	1	0	1	0	0	0	0	0	2	13.33	<0.0001	^∗∗∗∗^
SZWUSS186	10	1	9	0	0	0	0	0	0	10	66.67	0.0011	^∗∗^
SZWUSS202	Unknown	1	2	1	1	0	0	0	0	4	26.67	<0.0001	^∗∗∗∗^
SZWUSS203	Unknown	1	3	1	0	1	0	0	0	6	40.00	<0.0001	^∗∗∗∗^
SZWUSS204	Unknown	1	3	2	0	0	0	0	0	6	40.00	<0.0001	^∗∗∗∗^
SZWUSS225	3	5	7	0	0	0	0	0	0	12	80.00	0.0862	ns
SZWUSS233	11	0	0	1	0	0	0	0	1	2	13.33	<0.0001	^∗∗∗∗^

*Note:* “ns” indicates no significant difference.

^a^The survival outcomes of the zebrafish groups were compared with that of the highly pathogenic strain SC070731 using the log‐rank (Mantel‐Cox) test.

^∗^Indicates *p* <  0.05.

^∗∗^Indicates *p* <  0.01.

^∗∗∗∗^Indicates *p* <  0.0001.

**Table 4:** The results of the BALB/c mice infection experiment.

Strain	*cps* type	Deaths at different post‐infection time points							Total deaths	Mortality rate (%)	*p* value	Significance^a^
		1 d	2 d	3 d	4 d	5 d	6 d	7 d				
SC070731	2	10	0	0	0	0	0	0	10	100.00	—	—
PBS	—	0	0	0	0	0	0	0	0	0.00	—	—
SZWUSS179	16	0	0	0	0	0	0	0	0	0.00	<0.0001	^∗∗∗∗^
SZWUSS181	16	0	0	0	0	0	0	0	0	0.00	<0.0001	^∗∗∗∗^
SZWUSS183	1/2	8	0	0	0	0	0	0	8	80.00	0.1462	ns
SZWUSS225	3	4	3	0	0	0	0	0	7	70.00	0.0043	^∗∗^

*Note:* “ns” indicates no significant difference.

^a^The survival outcomes of the mouse groups were compared with that of the highly pathogenic strain SC070731 using the log‐rank (Mantel‐Cox) test.

^∗∗^Indicates *p* <  0.01.

^∗∗∗∗^Indicates *p* <  0.0001.

## 4. Discussion

The *recN* gene of SS [15], the *HPS_219690793* gene of GPS [18], and the *apxⅣ* gene of APP [19] are highly species‐specific genetic targets widely used in clinical and laboratory diagnostics [31–36]. Notably, Rao et al. [3] previously developed a pentaplex PCR assay capable of simultaneously detecting SS, GPS, and APP that relied on the *gdh* gene for SS and the *16SrRNA* gene for GPS and APP. However, the *16S rRNA* gene has limitations for species‐level identification of the GPS. The GPS can be divided into two phylogenetic clusters based on *16S rRNA* sequence heterogeneity, which prompted Angen et al. [37] to develop a multiplex PCR using three primers to address this issue [17, 37]. In addition, *gdh* is not a reliable target for the identification of *S. suis*. Previous studies have shown that some *S. suis* isolates could not be correctly identified using the *gdh*‐based PCR assay, whereas isolates from other *Streptococcus* species could be misidentified as *S. suis* when *gdh* was used as the target [38, 39]. Therefore, we developed a triplex real‐time PCR assay that simultaneously detects the three target pathogens using the aforementioned species‑specific genes. The assay demonstrated high specificity, sensitivity, and reproducibility. The primers and probes for SS and GPS exhibited high specificity with no cross‐reactivity to other species. For APP, BLAST analysis revealed that the primers and probe showed high similarity only to a single strain of *Actinobacillus lignieresii* (CCUG 22229), which has been reported to be isolated exclusively from ruminants and horses [40, 41] and therefore does not compromise detection in porcine samples.

The assay was applied to 228 tonsil samples collected from clinically healthy pigs in Jiangsu, Guangdong, Guangxi, and Hunan provinces. At the regional level, high overall positivity of at least one pathogen was observed in all four provinces, with rates of 94.57% (87/92) in Guangdong, 87.76% (86/98) in Jiangsu, 90.91% (30/33) in Guangxi, and 100% (5/5) in Hunan. The co‐detection rate for at least two pathogens was 44.57% (41/92) in Guangdong, 45.92% (45/98) in Jiangsu, 30.30% (10/33) in Guangxi, and 40.00% (2/5) in Hunan. Compared with previous reports from healthy pigs in eastern China, where Zhu et al. [5] found detection rates of 71.0% for SS, 55.6% for GPS, and 0.4% for APP and noted that co‐detections were more common than single detections, our data similarly show a predominance of SS and frequent SS‐GPS co‐detection in clinically healthy herds. However, studies based on diseased pigs have reported different patterns. For example, Rao et al. [3] reported higher APP positivity (42.47%) in symptomatic pigs in Guangxi, and Renzhammer et al. [14] summarized data from Austrian diseased pigs showing SS as the most common bacterium (30.6%), followed by APP (11.9%) and GPS (8.8%), with SS‐GPS being the dominant co‐detection pattern. Together with other regional surveys [7, 42–44], these findings indicate that SS, GPS, and APP are widely distributed globally, with SS‐GPS co‐detection recurrently reported as the most frequent combination, while the prevalence and co‐detection rates vary by region, health status, and other reasons.

Its reliable performance in complex clinical samples supports its utility for the rapid identification of mixed detections in clinical diagnostics. Notably, SS and GPS were successfully isolated from samples that were positive by the triplex assay but negative by conventional PCR, further supporting the practical reliability of the assay. However, no APP isolate was recovered. This failure is likely attributable to several factors. First, APP isolation generally requires fresh samples. In this study, samples were collected at the slaughterhouse and usually reached the laboratory for isolation after 1–2 days, which may have reduced the recovery rate. Second, the samples were obtained from the tonsils of clinically healthy pigs, in which the APP burden was likely low. Third, no suitable selective medium was used for APP isolation. Because tonsillar samples contain abundant commensal bacteria, the overgrowth of competing bacteria during enrichment may have further increased the difficulty of recovering APP.

Previous studies have established SS as a significant reservoir of antibiotic resistance genes [45]. In this study, all SS isolates recovered from triple‐positive samples were resistant to azithromycin, erythromycin, and tetracycline; more than half of the isolates also exhibited resistance to lincomycin and clindamycin; all isolates remained susceptible to amoxicillin, cefotaxime, rifampin, and vancomycin. This resistance profile aligns with patterns reported for SS in Thailand [46], Switzerland [47], Brazil [48], and Spain [49], as well as our earlier studies [27, 28, 50]. Notably, 91.67% of the isolates (11/12) were MDR. This high prevalence of MDR is likely linked to inappropriate antimicrobial use in veterinary practice, underscoring the urgent need to strengthen veterinary antimicrobial stewardship [51].

SS strains originating from clinically healthy pigs may represent an important reservoir for transmission to susceptible pigs and humans [52–54]. In this study, strain SZWUSS183 (*cps* type 1/2, ST7) exhibited a high level of virulence comparable to that of a well‐characterized highly virulent ST7 reference strain. Serotype 1/2 is structurally highly similar to the zoonotic serotype 2, differing by only a single sugar residue [55]. However, it has not been associated with human infections [56, 57]. ST7 is largely endemic to China and has been implicated in two historical human outbreaks [7], but it is predominantly linked to serotype 2. Therefore, the *cps* type 1/2‐ST7 combination identified here represents an atypical and uncommon genetic profile. This combination was first reported in diseased pigs from the Czech Republic and was considered to pose a potential epidemiological risk [58]. Furthermore, we screened strain SZWUSS183 for 26 putative zoonotic virulence factors of *S. suis* [59]. Of these, 19 genes were detected, including *cps2F*, *cps2B*, *cps2G*, *cps2J*, *cps2L*, *neuB*, *sp1*, *sly*, *rgg*, *sbp1*, *sbp2*, *hylA*, *rfeA*, *tran*, *pnuC*, *cbp40omp40*, *zmpC*, *fhb_2*, and *igdE*, suggesting that this strain has considerable pathogenic potential. Moreover, recent surveillance data from Italy suggest that the prevalence of serotype 1/2 may be underestimated due to the diagnostic cross‐reactivity with serotype 2 [57]. Together, the identification and virulence characterization of the *cps* type 1/2‐ST7 strain in this study not only document the presence of this rare profile but also underscore the importance of accurate molecular typing for surveillance and risk assessment of potentially pathogenic SS lineages.

Serotype 3 is increasingly recognized as an important cause of clinical disease in pigs globally [60, 61]. This is supported by surveillance data from 15 provinces in China, in which *cps* type 3 represented 12% of SS isolates from diseased swine, with all identified ST117 isolates belonging to *cps* type 3, further underscoring its clinical relevance [62]. The virulent strain SZWUSS225 isolated in this study also aligns with this serotype‐genotype profile. These data highlight the growing clinical relevance of serotype 3 strains, particularly those of the ST117 lineage.

In this study, samples were collected exclusively from the tonsils of healthy pigs. Thus, our findings may not fully reflect co‐infection patterns under clinical disease conditions. Although samples were obtained from four provinces, the geographic coverage was limited, which may affect the generalizability of the results. Future studies should expand sampling areas and include tissues from diseased pigs to more comprehensively assess the epidemiological features and clinical implications of SS, GPS, and APP co‐infections.

In summary, we developed a triplex real‐time PCR assay targeting the *recN* gene of SS, the *HPS_219690793* gene of GPS, and the *apxⅣ* gene of APP, enabling rapid, specific, and sensitive detection for bacterial co‐detections in pigs. The recovery of multidrug‑resistant SS isolates underscores the persistent challenge of antimicrobial resistance in swine production and calls for reinforced stewardship measures. Furthermore, the detection of an atypical, virulent *cps* type 1/2‑ST7 lineage from healthy carriers, alongside the widely circulating *cps* type 3‑ST117 profile, suggests the presence of potentially underestimated pathogenic lineages. Collectively, this study enhances diagnostic capacity and supports improved surveillance strategies for the prevention and control of respiratory bacterial co‐detection in pigs.

## Author Contributions

Data curation: Hongkun Zhuang and Zongfu Wu. Formal analysis: Hongkun Zhuang, Jinlu Zhu, and Zongfu Wu. Investigation: Hongkun Zhuang, Shun Kang, Chenxu Zheng, Zeren Peng, and Zongfu Wu. Project administration: Zongfu Wu. Supervision: Zongfu Wu. Writing – original draft: Hongkun Zhuang, Jinlu Zhu, and Zongfu Wu. Writing – review and editing: Hongkun Zhuang and Zongfu Wu.

## Funding

This work was supported by the National Key Research and Development Program of China (Grant 2023YFD1800503) and the Scientific Research Project of the General Administration of Customs, China (Grant 2024HK031).

## Conflicts of Interest

The authors declare no conflicts of interest.

## Supporting Information

Additional supporting information can be found online in the Supporting Information section.

## Supporting information


**Supporting Information** Table S1: Strains used in the study. Table S2: Primers of recombinant plasmid. Table S3: Optimal reaction system of the triplex assay. Table S4: LOD of the triplex assay. Table S5: Intra‐assay and inter‐assay reproducibility of the triplex assay. Table S6: Strains isolated from tonsil samples positive by triplex assay but negative by conventional PCR. Table S7: The result of MICs.

## Data Availability

The draft genome sequencing data have been uploaded into the NCBI database under the accession numbers listed in Table 2. Additional supporting information can be found in the Supporting Information section of this article.
