# Supplementary material for: A Triplex Real‐Time PCR Assay for Simultaneous Detection of Streptococcus suis, Glaesserella parasuis, and Actinobacillus pleuropneumoniae
Source: Transbound Emerg Dis. 2026 May 21;2026:9983141. doi: 10.1155/tbed/9983141 (PMC13195185; doi:10.1155/tbed/9983141)
Supplement: Supplementary file 1 — Supporting Information Table S1: Strains used in the study. Table S2: Primers of recombinant plasmid. Table S3: Optimal reaction system of the triplex assay. Table S4: LOD of the triplex assay. Table S5: Intra‐assay and inter‐assay reproducibility of the triplex assay. Table S6: Strains isolated from tonsil samples positive by triplex assay but negative by conventional PCR. Table S7: The result of MICs. [file TBED-2026-9983141-s001.docx]

**Supplemental Table 1.** Strains used in the study.

| **Strain** | **Species** | **Source** |
| --- | --- | --- |
| SC070713 | *Streptococcus suis* | Laboratory collection |
| WUGPS001 | *Glaesserella parasuis* | Laboratory collection |
| WUAPP001 | *Actinobacillus pleuropneumoniae* | Laboratory collection |
| WUSP097 | *Streptococcus pasteurianus* | Laboratory collection |
| ATCC29213 | *Staphylococcus aureus* | Laboratory collection |
| ATCC35246 | *Streptococcus equi* subsp. *zooepidemicus* | Laboratory collection |
| WUQT049 | *Streptococcus agalactiae* | Laboratory collection |
| WUQT019 | *Klebsiella pneumoniae* | Laboratory collection |
| WUQT022 | *Escherichia coli* | Laboratory collection |
| BM407 | *Streptococcus suis* | NCBI reference genome |
| YHP1818 | *Glaesserella parasuis* | NCBI reference genome |
| S4074 | *Actinobacillus pleuropneumoniae* | NCBI reference genome |

**Supplemental Table 2.** Primers of recombinant plasmid.

| **Primers** | **( 5’→3’ ) Sequence** | **Size (bp)** |
| --- | --- | --- |
| SS-pMD19-T-F | CTACAAACAGCTCTCTTCT | 336 |
| SS- pMD19-T-R | ACAACAGCCAATTCATGGCGTGATT |  |
| GPS- pMD19-T-F | ACAACCTGCAAGTACTTATCGGGAT | 275 |
| GPS- pMD19-T-R | TAGCCTCCTGTCTGATATTCCCACG |  |
| APP- pET-28a-F | CAGCAAATGGGTCGCGGATCCAAAATGGCAGGCAATAATTCTCG | 194 |
| APP- pET-28a-R | TTGTCGACGGAGCTCGAATTCCAACAACGTCGCACAATTAATCTA |  |

**Supplemental Table 3.** Optimal reaction system of the triplex assay.

| **Reagent** | **Volume (μL)** |
| --- | --- |
| Fluorescent PCR Enzyme Master Mix (Probe Method) | 15 |
| SS-F(10μM) | 0.8 |
| SS-R(10μM) | 0.8 |
| SS-P(10μM) | 0.4 |
| GPS-F(10μM) | 0.4 |
| GPS-R(10μM) | 0.4 |
| GPS-P(10μM) | 0.2 |
| APP-F(10μM) | 0.8 |
| APP-R(10μM) | 0.8 |
| APP-P(10μM) | 0.4 |
| Template DNA | 5 |
| Total | 25 |

**Supplemental Table 4.** LOD of the triplex assay.

| **Template concentration**  **(copies/μL)** | | **Total**  **Samples** | **Positive**  **Detection** | **Positive**  **Rate (%)** | **95% Confidence Region** |
| --- | --- | --- | --- | --- | --- |
| *S. suis* | 10^2^ | 20 | 20 | 100 | YES |
|  | 10^1^ | 20 | 14 | 70 | NO |
|  | 10^0^ | 20 | 0 | 0 | NO |
| *G. parasuis* | 10^2^ | 20 | 20 | 100 | YES |
|  | 10^1^ | 20 | 12 | 60 | NO |
|  | 10^0^ | 20 | 0 | 0 | NO |
| *A. pleuropneumoniae* | 10^2^ | 20 | 20 | 100 | YES |
|  | 10^1^ | 20 | 16 | 80 | NO |
|  | 10^0^ | 20 | 0 | 0 | NO |

**Supplemental Table 5.** Intra-assay and inter-assay reproducibility of the triplex assay.

| **Template concentration (copies/μL)** | | **Intra-assay repeatability test** | | **Inter-assay repeatability test** | |
| --- | --- | --- | --- | --- | --- |
|  |  | **Mean ± SD** | **CV %** | **Mean ± SD** | **CV %** |
| *S. suis* | 10^7^ | 18.866 ± 0.218 | 1.16 | 18.537 ± 0.294 | 1.58 |
|  | 10^6^ | 22.177 ± 0.270 | 1.22 | 21.845 ± 0.338 | 1.55 |
|  | 10^5^ | 25.100 ± 0.295 | 1.17 | 24.849 ± 0.217 | 0.88 |
|  | 10^4^ | 28.740 ± 0.174 | 0.60 | 28.356 ± 0.333 | 1.17 |
| *G. parasuis* | 10^7^ | 17.670 ± 0.318 | 1.80 | 17.746 ± 0.128 | 0.72 |
|  | 10^6^ | 21.126 ± 0.290 | 1.37 | 21.012 ± 0.101 | 0.48 |
|  | 10^5^ | 24.131 ± 0.268 | 1.11 | 24.278 ± 0.135 | 0.55 |
|  | 10^4^ | 27.621 ± 0.266 | 0.96 | 27.890 ± 0.333 | 1.17 |
| *A. pleuropneumoniae* | 10^7^ | 17.830 ± 0.362 | 2.03 | 17.948 ± 0.110 | 0.61 |
|  | 10^6^ | 21.290 ± 0.161 | 0.76 | 21.346 ± 0.164 | 0.77 |
|  | 10^5^ | 24.203 ± 0.271 | 1.17 | 24.444 ± 0.212 | 0.87 |
|  | 10^4^ | 27.681 ± 0.388 | 1.40 | 28.073 ± 0.346 | 1.23 |

**Supplemental Table 6.** Strains isolated from tonsil samples positive by triplex assay but negative by conventional PCR.

| **Strains** | **Species** | **Location** |
| --- | --- | --- |
| KSWUSS002 | *S. suis* | Jiangsu province |
| KSWUSS011 | *S. suis* | Jiangsu province |
| KSWUSS012 | *S. suis* | Jiangsu province |
| KSWUSS025 | *S. suis* | Hunan province |
| KSWUSS031 | *S. suis* | Jiangsu province |
| KSWUSS038 | *S. suis* | Jiangsu province |
| WUGPS008 | *G. parasuis* | Jiangsu province |
| WUGPS009 | *G. parasuis* | Jiangsu province |

**Supplemental Table 7.** The result of MICs.

| **Strains** | **Tested antimicrobial agents and breakpoints for resistant (µg/mL)** | | | | | | | | |
| --- | --- | --- | --- | --- | --- | --- | --- | --- | --- |
|  | **Penicillin**  ≥ 1 | **Amoxicillin**  ˃ 2 | **Cefotaxime**  ≥ 8 | **Rifampin**  ≥ 4 | **Vancomycin**  ˃ 1 | **Linezolid**  ˃ 2 | **Enrofloxacin**  ≥ 2 | **Chloramphenicol**  ≥ 16 | **Florfenicol**  ≥ 8 |
|  | **MIC value of *S. suis* strains (µg/mL)** | | | | | | | | |
| SZWUSS179 | 4 | 2 | 4 | ≤0.5 | ≤0.5 | 4 | 8 | 16 | 8 |
| SZWUSS181 | ≤0.5 | ≤0.5 | ≤0.5 | ≤0.5 | ≤0.5 | 2 | 8 | 8 | 16 |
| SZWUSS183 | ≤0.5 | ≤0.5 | ≤0.5 | ≤0.5 | ≤0.5 | ≤0.5 | ≤0.5 | 2 | 2 |
| SZWUSS184 | 8 | ≤0.5 | 1 | ≤0.5 | ≤0.5 | 4 | ≤0.5 | 8 | 16 |
| SZWUSS185 | ≤0.5 | ≤0.5 | ≤0.5 | ≤0.5 | ≤0.5 | ≤0.5 | ≤0.5 | 2 | 2 |
| SZWUSS186 | ≤0.5 | ≤0.5 | ≤0.5 | ≤0.5 | ≤0.5 | ≤0.5 | ≤0.5 | 4 | 8 |
| SZWUSS202 | ≤0.5 | ≤0.5 | ≤0.5 | ≤0.5 | ≤0.5 | ≤0.5 | ≤0.5 | 4 | 1 |
| SZWUSS203 | ≤0.5 | ≤0.5 | ≤0.5 | ≤0.5 | ≤0.5 | ≤0.5 | ≤0.5 | 2 | ≤0.5 |
| SZWUSS204 | ≤0.5 | ≤0.5 | ≤0.5 | ≤0.5 | ≤0.5 | ≤0.5 | ≤0.5 | 2 | 1 |
| SZWUSS225 | ≤0.5 | ≤0.5 | ≤0.5 | ≤0.5 | ≤0.5 | ≤0.5 | ≤0.5 | 2 | 2 |
| SZWUSS233 | ≤0.5 | ≤0.5 | ≤0.5 | ≤0.5 | ≤0.5 | 2 | 8 | 4 | 32 |
| SZWUSS234 | ≤0.5 | ≤0.5 | ≤0.5 | ≤0.5 | ≤0.5 | 1 | ≤0.5 | 4 | 1 |

**Supplemental Table 7 continued**

| **Strains** | **Tested antimicrobial agents and breakpoints for resistant (µg/mL)** | | | | | | | |
| --- | --- | --- | --- | --- | --- | --- | --- | --- |
|  | **Lincomycin**  ≥ 1 | **Clindamycin**  ≥ 1 | **Tiamulin**  ≥ 32 | **Gentamycin**  ≥ 16 | **Spectinomycin**  ≥ 128 | **Erythromycin**  ≥ 1 | **Azithromycin**  ≥ 2 | **Tetracycline**  ˃ 8 |
|  | **MIC value of *S. suis* strains (µg/mL)** | | | | | | | |
| SZWUSS179 | 256 | 256 | 32 | 256 | ＞256 | ＞256 | ＞256 | 128 |
| SZWUSS181 | 256 | 256 | 32 | 64 | 8 | ＞256 | ＞256 | 64 |
| SZWUSS183 | ＞256 | 256 | ≤0.5 | 4 | 16 | 32 | 32 | 32 |
| SZWUSS184 | ＞256 | 256 | 64 | 1 | 8 | ＞256 | ＞256 | 64 |
| SZWUSS185 | ＞256 | 256 | ≤0.5 | 64 | 16 | ＞256 | ＞256 | 64 |
| SZWUSS186 | ＞256 | ＞256 | 64 | 8 | ＞256 | 16 | 256 | 64 |
| SZWUSS202 | ＞256 | 256 | 64 | 1 | 8 | 128 | 64 | 32 |
| SZWUSS203 | ＞256 | 256 | 16 | 2 | 8 | 4 | 4 | 64 |
| SZWUSS204 | ＞256 | 256 | 64 | ≤0.5 | 8 | 256 | 256 | 64 |
| SZWUSS225 | ≤0.5 | ＞256 | ≤0.5 | 2 | 16 | 32 | 64 | 64 |
| SZWUSS233 | 1 | 256 | 16 | 2 | 16 | ＞256 | ＞256 | 64 |
| SZWUSS234 | ≤0.5 | 256 | 8 | ＞256 | 16 | ＞256 | ＞256 | 128 |
